# Rapid development and rupture of a pulmonary cyst in the early postoperative period after pulmonary resection: A case report

**DOI:** 10.1111/1759-7714.13421

**Published:** 2020-04-04

**Authors:** Nobutaka Kawamoto, Riki Okita, Masataro Hayashi, Masashi Furukawa, Hidetoshi Inokawa, Kazunori Okabe

**Affiliations:** ^1^ Department of Thoracic Surgery National Hospital Organization YamaguchiUbe Medical Center Ube Japan

**Keywords:** Giant bulla, postoperative air leak, postoperative complication, pulmonary cyst

## Abstract

Air leakage is a common complication after pulmonary resection, which is usually caused by direct lung damage during surgery. Herein, we describe a case in which a pulmonary cyst developed rapidly in the right lower lobe and ruptured 10 days after right upper lobectomy. A 49‐year‐old man, who was a heavy smoker, underwent thoracoscopic right upper lobectomy for primary lung cancer. No air leakage was observed postoperatively, and the chest drain tube was removed on postoperative day 1. Although his postoperative course was uneventful for more than a week, extensive subcutaneous emphysema developed unexpectedly on postoperative day 10. Computed tomography (CT) scan revealed a large pulmonary cyst in the right lower lobe that was not present before the right upper lobectomy. Surgery was performed on postoperative day 13, and it revealed a large thick‐walled pulmonary cyst in the right lower lobe. The cyst was filled with blood clots, and air leaks were observed inside it, suggesting that the dissection of the pulmonary parenchyma caused its development. The cyst wall was sutured together with the pulmonary parenchyma, and no air leakage was subsequently observed.

**Key points:**

**Significant findings of the study**

- In patients with fragile pulmonary tissue, the pulmonary parenchyma may become dissociated after pulmonary resection and induce rapid development of a pulmonary cyst.
- Risk factors for pulmonary cyst development include upper lobectomy and emphysema. Pulmonary cysts are often formed in the lower lobe.

**What this study adds**

- In patients with pulmonary emphysema post‐upper lobectomy, the fragility of the pulmonary parenchyma and hyperinflation of the remaining lung may cause dissection of the pulmonary parenchyma, resulting in massive air leakage.

## Introduction

Air leakage is a common complication after pulmonary resection, and it reportedly occurs in approximately 8%–26% of patients who undergo pulmonary lobectomy.^1^ Although air leaks can be successfully resolved by thoracic drainage alone in most cases, prolonged air leakage requires additional treatment, such as pleurodesis, reoperation, and/or bronchial embolization, in some cases.^1^, ^2^, ^3^ Air leaks are usually caused by direct lung damage during a surgical procedure. Herein, we describe a case of postoperative air leakage caused by a pulmonary cyst that developed rapidly and ruptured 10 days after pulmonary resection.

## Case report

**Figure 1:**
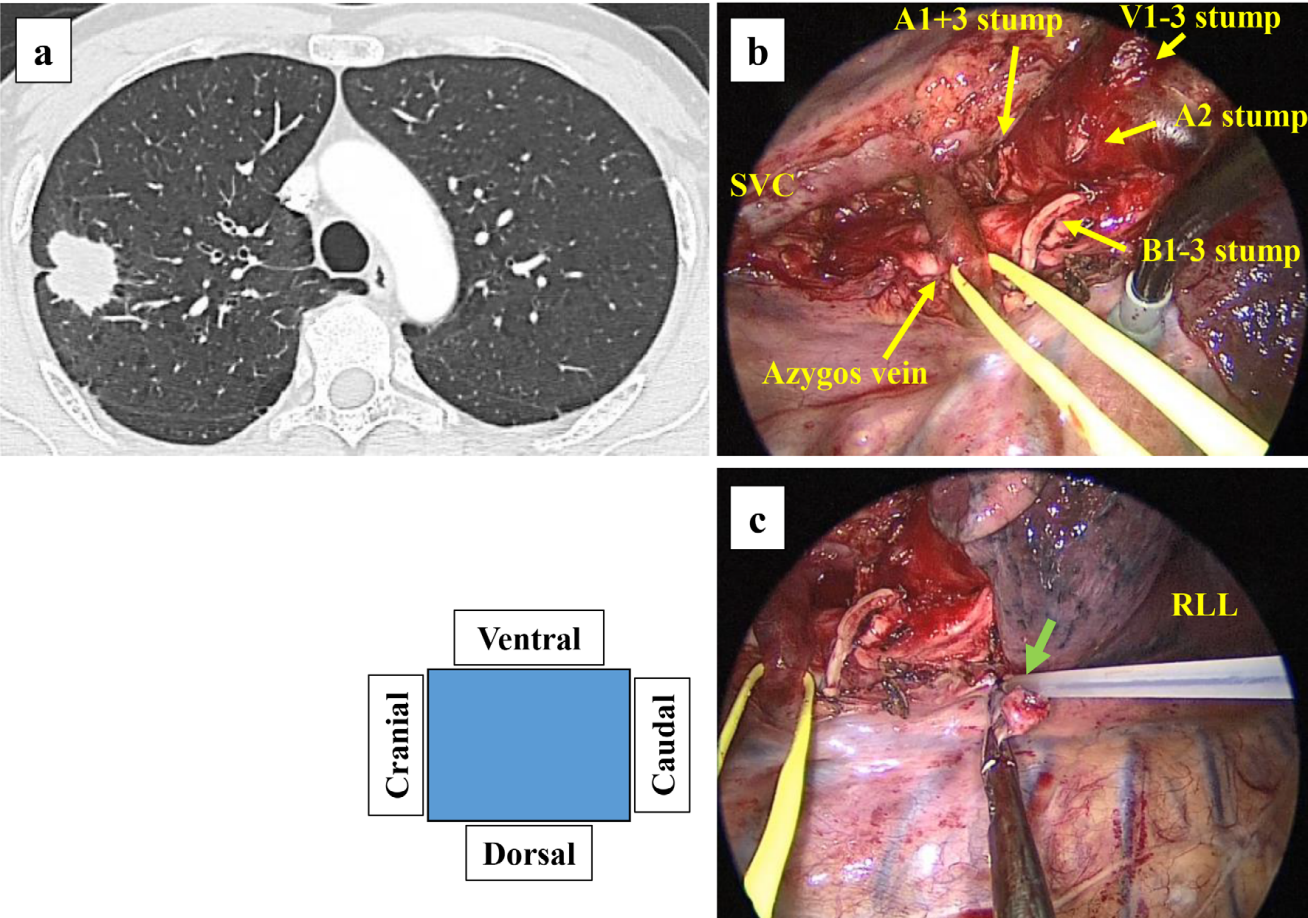
Preoperative computed tomography image and surgical findings of right upper lobectomy. (**a**) Computed tomography (CT) scan revealed lung cancer in the right upper lobe and emphysema mainly in the bilateral upper lobes. (**b**) Surgical findings after right upper lobectomy. (**c**) A pulmonary cyst at the top of the right sixth segment was ligated (green arrow). RLL, right lower lobe; SVC, superior vena cava.

Chest radiography of a 49‐year‐old man with a history of 62 pack‐years of smoking revealed a pulmonary nodule. Spirometry indicated a forced expiratory volume of 3.15 L (63.9%) in the first second and an obstructive ventilation disorder. In laboratory tests, the tumor marker carcinoembryonic antigen was 9.1 ng/mL (<5.0 ng/mL). Computed tomography (CT) scan revealed a 3.3 cm lung tumor in the right upper lobe and emphysema mainly in the bilateral upper lobes (Fig 1a). No pulmonary cysts were evident in the interlobar area of the right lower lobe. Thoracoscopic surgery was performed based on suspected right upper lung cancer at clinical stage IB.

The intraoperative frozen section diagnosis was adenocarcinoma, and right upper lobectomy and mediastinal lymph node dissection (ND2a‐1) were subsequently performed (Fig 1b). The upper and lower lobes were observed to be completely separated; thus, no further separation of the two lobes was required. No pulmonary cysts were detected in the interlobar surface of the lower lobe. A small pulmonary cyst was detected at the top of the right sixth segment, which was ligated (Fig 1c). The surgical field was secured by compression technique alone, without grasping the part of the lungs that did not require resection. A 20 Fr chest drain tube was placed in the thoracic cavity, and the suction pressure was set to 5 cmH_2_O. No air leakage was observed postoperatively, and the chest drain tube was removed on postoperative day (POD) 1.

**Figure 2:**
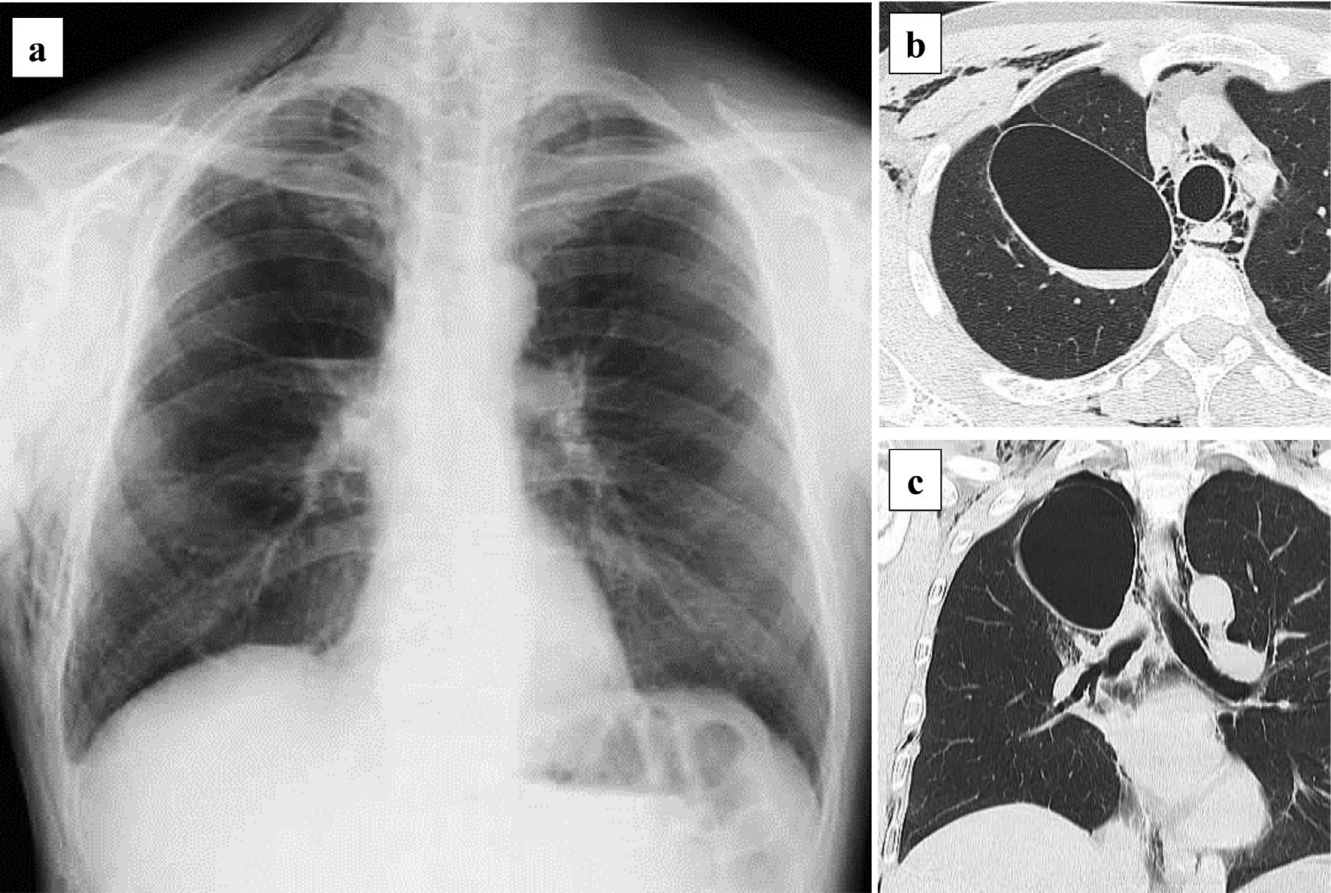
Imaging of the pulmonary cyst that developed rapidly. (**a**) Chest radiography depicting extensive subcutaneous emphysema, suggesting a pulmonary cyst in the upper right lung field. (**b**, **c**) Computed tomography depicting subcutaneous emphysema and mediastinal emphysema and an 8 cm fluid‐filled pulmonary cyst in the right lower lobe.

**Figure 3:**
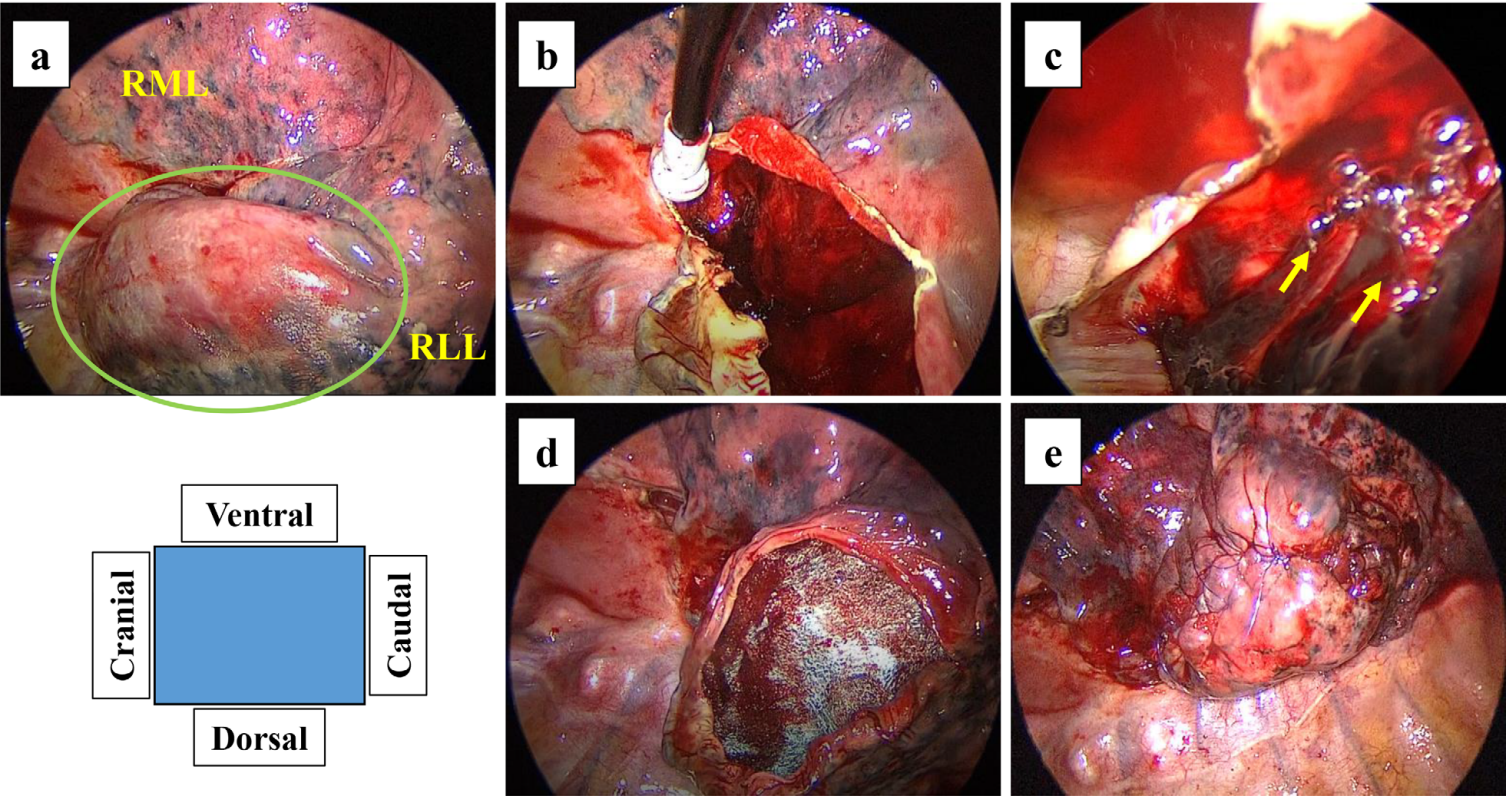
Surgical findings of a pulmonary cyst that developed rapidly and ruptured. (**a**) A large pulmonary cyst is observed in the right lower lobe (green circle). (**b**, **c**) Blood clots and multiple air leaks are evident inside the cyst (yellow arrows: air leaks). (**d**) Fibrin glue and a polyglycolic acid sheet were applied inside the cyst. (**e**) The cyst wall was sutured together with the pulmonary parenchyma. RLL, right lower lobe; RML, right middle lobe.

**Figure 4:**
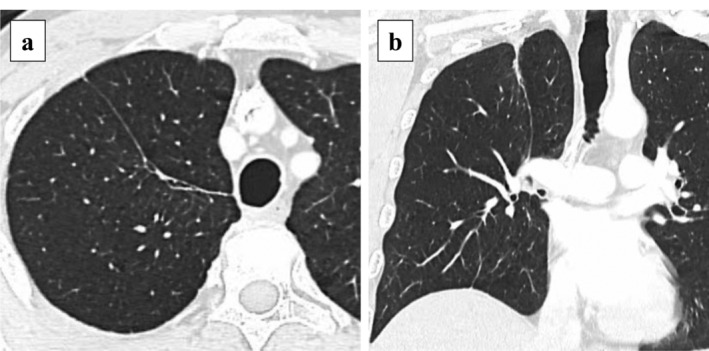
Computed tomography findings at the nine month follow‐up examination after surgical treatment of the secondary pulmonary cyst. (**a**, **b**) No new pulmonary cysts had developed.

The patient was scheduled for discharge on POD 10, but on that day, extensive subcutaneous emphysema suddenly developed. Chest radiography and CT revealed a large new pulmonary cyst in the right lower lobe, which developed after the right upper lobectomy (Fig 2). Surgery was performed on POD 13 due to suspected rupture of the new cyst. A broad‐based thick‐walled cystic lesion was noted in the interlobar surface of the lower lobe (Fig 3a). This newly formed cyst had developed far from the previously ligated cyst. Blood clots and multiple air leaks were detected inside the new cyst (Fig 3b,c). After removing the blood clots, fibrin glue and a polyglycolic acid sheet were applied inside the cyst (Fig 3d), and the cyst wall was sutured together with the pulmonary parenchyma (Fig 3e). No air leakage was observed postoperatively, and the chest drain tube was removed on POD 3. The lung adenocarcinoma was stage IB on pathological diagnosis, and tegafur‐uracil was administrated as adjuvant chemotherapy. At nine‐month follow‐up, no new pulmonary cysts had developed (Fig 4).

## Discussion

Rapid development of new pulmonary cysts in the early postoperative period are very rare.^4^ The check valve is an important mechanism of bulla formation, whereby air is progressively trapped in alveoli distal to the obstruction,^5^ resulting in the formation of various sized pulmonary cysts in the lung. The walls of giant bullae are generally thin and translucent, and many trabecula‐like structures exist inside giant bullae.^6^ In our case, the cyst wall was very thick and full of blood clots, suggesting that the cystic lesion had formed via dissociation of the pulmonary parenchyma.

A review of reported cases suggests that the above‐described complication mainly occurs in patients with emphysema post‐upper lobectomy and that cysts generally form in the lower lobe.^4^ The interlobar surface area is widest in the lower lobe of the right lung. These facts lead to the hypothesis that post‐upper lobectomy, the lower lobe is hyperinflated due to increased negative pressure in the thoracic cavity; thus, if the pulmonary parenchyma is fragile due to emphysema, this hyperinflation may cause pulmonary parenchyma dissection.

The above‐described complication is very rare, but it may occur in emphysema patients who have undergone pulmonary resection of the upper lobe.

## Disclosure

None of the authors have any potential conflicts of interest associated with this report.
